# Hominid sexual nature

**DOI:** 10.1007/s12064-020-00312-8

**Published:** 2020-03-13

**Authors:** Christopher Mogielnicki, Katherine Pearl

**Affiliations:** 1 Bialystok University of Technology, Bialystok, Poland; 2 Bialystok, Poland

**Keywords:** Hominid sexuality, Hominid social structures, Sexual jealousy, Mate choice

## Abstract

The aim of the paper is to identify psychosomatic evolutionary adaptations of hominids, which direct them at maximizing their reproductive success, and on the basis of which their various social structures are built. Selected features of the hominid last common ancestor were extracted; by reducing the influence of the social structure, they were defined as the hominid “sexual nature”; these considerations were supported by the analysis of sexual jealousy as a function of socio-environmental conditions. The “sexuality core” of a hominid female was defined as “selective polyandry”—the female selects the best males among those available; and of a hominid male as “tolerant promiscuity”—the male strives for multi-male and multi-female copulations with sexually attractive females. The extracted “sexuality cores” condemn hominids to a patriarchal social structure and thus to sexual coercion and jealousy. The source of male sexual jealousy is limited access to females. Hominid female jealousy of the male results mainly from the need for protection and support. Hominids’ social structures are determined by females’ sexual selectivity or opportunism and by their continuous or periodic proceptivity and estrus signaling. Evolutionary functions developed by women: out-estrus sexuality, copulation calls, multiple orgasms, allow them to obtain the best possible spermatozoid. The institution of marriage blocks the influence of sexual selection in the species *Homo sapiens*.

## Introduction

Due to the cultural multiplicity of reproduction systems in the species *Homo sapiens*, it is difficult to clearly determine the type of its breeding strategy. This demonstrates the flexibility and adaptability of the reproduction system that depends on environmental and social conditions. Human reproductive systems can generally be divided into patriarchal and matricentric (matrifocal). A vast majority of human societies function under patriarchal conditions, which include polygyny (where one man can have several wives), a serial monogamy (where a man can have only one wife), and polyandry (where one woman has several husbands). In matricentric structures, in turn, marriages do not occur at all.

According to the Trivers (1972) theory, a gender that invests more in the offspring will be more careful when choosing a partner, while the gender that invests less will compete for him/her. The behavior of genetically close relatives of humans, common chimpanzees (*Pan troglodytes,* hereafter also called chimpanzees), correlates with this theory. Females, making 100% of parental investments, receive food from males and suffer sexual aggression from them (Goodall 1986). On the other hand, bonobo chimpanzees (*Pan paniscus,* hereafter also called bonobos) have free sexual access to one another and the females, who also bear full parental investments, are not particular about choosing a partner but have priority in access to food (Stanford 1998). The diversity of reproductive strategies, not only in the species *Homo sapiens*, but also among the entire family of hominids, tends to search for a “common sexual denominator” for all hominids—the hominid “sexual nature.”

Hominid (*Hominidae*) is a family of great apes that includes species whose representatives are the largest among the primates. They show intelligence, tendencies to adopt a bipedal attitude, and the ability to produce and use tools. This paper describes a monophyletic group of the hominid species: the gorilla (*Gorilla,* hereafter also called gorilla), common chimpanzee (*Pan troglodytes*), bonobo (*Pan paniscus*) and modern human *Homo sapiens.* They are all social species. Apart from chimpanzee, bonobo and gorilla, *Homo sapiens’* close relative also includes the orangutan (*Pongo*). These species, together with extinct human forms, form the *Hominidae* family (Wilson and Reeder 2005). In this study, the orangutan is not analyzed because of belonging to the subfamily *Ponginae*, a more distant kinship with humans and its solitary way of life (Locke et al. 2011).

Gorillas function in the terrestrial environment, in homogenic harem groups (a male and his females), in which daughters and sons leave the family. Gorilla females do not exhibit external signs of ovulation, and therefore, an estrus is signaled by behavioral signals such as proceptive behavior (Stewart 1977; Watts 1991). Chimpanzees live in large terrestrial and arboreal heterogeneous herds with a dominant male, and their females signal the estrus with anal–genital swelling (Goodall 1986). Matriarchal bonobos are also terrestrial and arboreal hominids which live in heterogeneous herds. They do not have the dominant male; males are dominated by females. Bonobo females simulate a permanent state of ovulation (Stanford 1998). Promiscuity and constant receptivity of the bonobo females caused the disappearance of the need to compete for the access to them and thus the disappearance of the dominant male institution. The lack of the need to compete for the females, as a result of the abundance of sex, is anatomically manifested in bonobos by the reduction of canine dimorphism (Wrangham and Peterson 1996; De Waal and Lanting 1997).

*Homo sapiens* is definitely dominated by militant, patriarchal social structures with hierarchized males (Otterbein 1970; Divale 1972; Keeley 1996). It was found that residence patterns are related to the frequency of violence between groups: communities based on related women are characterized by a lower rate of conflicts than patrilocal ones (Adams 1983). The same qualitative observation was made in a comparative study of humans and primates (Manson et al. 1991). *Homo sapiens* is terrestrial and can form social structures based on both related males and females. Its females, just like bonobos, also signal the permanent state of the estrus. The females of all *Hominidae* species copulate also out of an estrus season (Savage-Rumbaugh and Wilkerson 1978; Stoinski et al. 2009a, b).

On the basis of selected features of the extant hominid species mentioned above, analogical features of their last common ancestor (LCA)—*Hominidae* clad, have been determined. To determine LCA features, based on phylogenetic relationships of living hominids presented in Scally et al. (2012) and Prüfer et al. (2012), the cladistic systematic analysis method was used (Hennig 1965). By reducing the influence of the LCA social structure on the features of its representatives, the “sexuality cores” of the hominid male and female have been designated. In this paper the “sexuality core” will be, independently of the social structure, biological and psychological dispositions, on the basis of which hominids’ social structures are created as a function of environmental conditions. In other words, “sexuality cores” are constant in the course of evolution, common for four hominid species and independent of the environmental influences; they are male and female sexual functions that maximize the hominids’ reproductive success. To determine these “cores,” the expected levels of sexual jealousy of the hominid male and female, depending on the access to sexual partners and to environmental resources, have also been designated.

## Female mating predispositions

Sexual behavior during infertile periods (including infertility before entering into the reproductive period) is not uncommon among apes. It occurs, e.g., in order to obtain food from a male or to reconcile among bonobos (De Waal 1987). However, Furuichi (1987) found that although bonobo females copulate when they are not maximally swollen, more than 95% of matings were observed during maximal or near-maximal swelling. It is the same for chimpanzees—97% (Goodall 1986). As for sexual receptivity, bonobo females are more flexible than other apes. It became a fundamental premise of bonobo sexuality and of the bonobo’s link to human behavior because, among the primates, only bonobo and human females are sexually active outside of the periovulatory period (Stanford 1998).

Swollen chimpanzee females copulate with multiple males during the early stages of their swelling cycle. As many as 50 copulation bouts with eight males during 1 day were observed and the swollen females copulated with up to eight adult males within several minutes (Goodall 1986). Bonobo females actively solicit sex from a range of males and may copulate multiple times per hour while swollen (Kano 1992). Gorilla females usually tend to mate only for a few days during a 28-day cycle (probably at the time of ovulation), but they also show uncommon periods of proceptivity at other times (Harcourt et al. 1980).

Female copulatory vocalizations, i.e., sounds issued by a female before, during, or just after a sexual intercourse, common to many primates (including the humans) are strongly associated with a promiscuous reproductive system. Females from promiscuous species emit more complex copulation calls than the females from polygynous or monogamous species. The degree of promiscuity of the females allows to predict how they tend to vocalize during the copulation. It is believed that copulatory vocalizations have two basic functions: to encourage other males to mate and to increase the protection of the act by a copulating male (Pradhan et al. 2006).

The analysis of the copulation calls of yellow baboon females (*Papio cynocephalus*) showed that these sounds inform both about the condition of the female (the closer the female is to the ovulation, the more complicated sounds) and the social status of the male copulating with her (the higher the male in the hierarchy, the longer the scream consisting of a larger number of acoustic units) (Semple 2001). The primate females during the copulation let out shouts that cannot be heard in any other situation. An orgasm or symptoms of sexual pleasure are easily recognized (Small 1989). According to Small, macaque wander (*Macaca silenus*) females attract by such calls out-group males to differentiate the genotype in the population. In Barbary macaques (*Macaca sylvanus*), the likelihood of male ejaculation is directly related to the intensity of female vocalizations (Todt and Pohl 1984) and this appears to be independent of the male’s copulatory effort (Pfefferle et al. 2008). The copulation calls are also present in the chimpanzee (Hauser 1990; Townsend et al. 2008), bonobo (Clay et al. 2011), gorillas (Nadler 1976) and *Homo sapiens*. Adaptation of vocalization evolved and persisted in both arboreal (the chimpanzees) and terrestrial (baboons) species, despite the threats that it carried.

Matsumoto-Oda (1999) reports that during an earlier estrus phase, the chimpanzee females copulate quite freely with many males. However, in the later stage of the estrus, when the probability of fertilization is the highest, they copulate rather with high-ranking adult males. A hormonal analysis carried out by Townsend et al. (2008) showed that the chimpanzee females vocalized much more often when they were with high-ranking males and they suppressed the calls when there were high-ranking males nearby. According to the authors, the copulatory vocalizations can thus be one of the potential strategies used by the female to advertise receptivity to the high-ranking males, to confuse fatherhood and to provide future support from these socially important individuals. In the case of women, it was also recognized that at least one element of these responses was under conscious control, providing them with an opportunity to manipulate the male behavior, e.g., to accelerate partner’s orgasm, when a woman was already weary (Brewer and Hendrie 2011).

Although all mammal females have a clitoris, there was a conviction confirmed by zoologists’ research that most animals, with the exception of the bonobos, chimpanzees (Allen and Lemmon 1981; Wrangham 1993) and macaques (*Macaca arctoides*) (Chevalier-Skolnikoff 1974) do not feel an orgasm. However, Troisi and Carosi (1998) note that it is possible to cause the orgasm in every primate female through artificial stimulation. Having examined the sexuality of the primates, Dixson (1998) states that the primate females, especially those from promiscuous reproductive systems (such as the macaques or chimpanzees), have the orgasms while copulating. In contrast, the females of mostly monogamous gibbons (*Hylobatidae*) or harem gorillas do not show any visible signs of the orgasm (Dixson 1998).

In the case of the women, an orgasmic phase can occur repeatedly within a single sexual intercourse. An average woman with optimal stimulation is able to achieve three to five orgasms caused by manual stimulation (Masters and Johnson 1966). The women do not enter, as it is the case of men, into the refraction phase as a result of achieving the orgasm. For some time they remain at a high level of excitement, which slowly decreases, and in some women even very slowly. During this period, the woman subjected to erotic stimuli can easily achieve a greater number of the orgasms. Studies of mechanical stimulation of the pubic mound using a vibrator showed that orgasmic sensations were intense and achieved in a minimum of time one after another in the waves of the multiple orgasms, closing a single sexual episode (Maers and Johnson 1966).

Basson (2001) indicates that some women, during an ovulatory period or after prolonged sexual abstinence, have a kind of “sexual hunger”—the intensification of sexual needs, which is called a “spontaneous desire,” and which is related to the action of hormones (including androgens), dopamine, oxytocin, noradrenergic receptors in the central nervous system or structures in the limbic system responsible for emotions. This state increases the susceptibility to sexual stimuli, and the rate and intensity of stimulation. Basson believes that in addition to these cases, the women’s desire has a psychological background and results from the need for tenderness, strengthening ties or recognition of their own attractiveness. Only such a motivated woman opens herself to sexual stimulation and as a result, the woman gets the ability to generate excitement. Basson calls this desire the “triggered desire,” or responding to the stimulus.

An ability to achieve the orgasm by the women depends on the phase of a monthly cycle: estrogens stimulate sexual tendencies (affect the blood supply to the vagina, labia and clitoris, and the hydration of the vagina), while the progesterone suppresses them. Androgens, in turn, increase sexual activity. The testosterone regulates the level of excitement and desire, and its concentration increases markedly a few days before ovulation. The sexual fitness and attractiveness of the women reach the highest level in the ovulatory period (Rowland 2006; Bullivant et al. 2004). In this period, the women have the orgasm most easily (Wallen and Lloyd 2011; Azadzoi and Siroky 2010; Dunn, et al. 2005). During the ovulatory period, the women have sexual relations more than three times more often than during the postovulatory phase (Baker and Bellis 2014).

Evolutionary adaptations in the form of clear ovulation signaling, the presence of multiple orgasms and the female copulation calls predispose species to multi-male mating. It is also known that in the case of the women, adapting to the conditions of the multi-male mating is also indicated by: the ability of the female reproductive system to assess immune compatibility of sperm on the basis of its chemical structure (Birkhead and Pizzari 2002; Barratt et al. 2009), preference of the reproductive system for the sperm delivered during the orgasm (Baker and Bellis 1993b). In *Homo sapiens*, additional factors are male evolutionary adaptations to sperm competition, e.g., testis size (Møller 1988), ejaculate adjustment (Baker and Bellis 1993a; Shackelford et al. 2002), sexual arousal (men’s sexual fantasies often involve multiple partners) (Pound 2002), ejaculate gametes’ role (Baker and Bellis 1988; 1989) and penis shape (Gallup et al. 2003; Mautz, et al. 2013).

## Male–female food transfer

Evolutionary sexual conditions of *Homo sapiens* indicate a high degree of mismatch between the women and men in terms of long-term satisfaction of mutual needs (Table 1).

**Table 1:** Sexual conditioning of *Homo sapiens* in the context of monogamy

Woman	Man
“Triggered” out-estrus sexuality, “sexual hunger” during the ovulatory period (Basson 2001)	Permanent sexual readiness, the Coolidge effect (Kinsey et al. 1948; Symons 1979; James 1981)
Long vaginal penetration necessary to achieve the orgasm (Weiss and Brody 2009)	Short copulation time (Kinsey et al. 1948; Waldinger et al. 2006)
Tendency to multiple orgasms (Masters and Johnson 1966)	Refraction after the orgasm

A basic “common interest” that would combine the *Homo sapiens* male and female, and which would evolutionarily enforce monogamous behavior, would be collaborative long-term offspring care. However, great ape males are not monogamous and do not bear paternal investments; the quality of offspring is measured by the mother’s ability to provide resources (Kramer 2011). Modern men also avoid, if possible, paternal investments. Therefore, as a rule, the burden of carrying pregnancy and feeding the young rests on the hominid females. Thus, the female who will bear the entirety of the parental investments will expect support from the males with whom she has sexual relations. The phenomenon of male–female food sharing occurs in all species of the analyzed apes, despite the diversity of their social structures.

Several hypotheses based on a reciprocity rule suggest social benefits of sharing food in the chimpanzees: “food for sex,” “food for grooming” and “food for support” (Stanford et al. 1994; De Waal 1997; Mitani and Watts 2001). These hypotheses are not mutually exclusive (Crick et al. 2013). A comparative study showed that males gave priority to access to food they possessed to females in the estrus, especially to those they had recently copulated with (Yerkes 1941). A long-term study conducted by Gomes and Boesch (2009) implied that among the chimpanzees, “food for sex” is not a short-term food exchange aimed at copulation, but a long-term strategy for building relationships that enable future mating.

Many similarities in the food sharing are seen between the chimpanzee and the bonobos that live wild (Kano 1980). Jaeggi et al. (2013) show, however, that the bonobos use seduction and sociosexual contacts to reduce tension caused by monopolized food, which is not shown by the chimpanzees. Food transfer was not observed in wild mountain gorillas (*G. beringei*) (Schaller 1963) and lowland gorillas (*G. G. Gorilla*) (Dixson 1981). However, it was reported that a lowland gorilla male kept in captivity moved a piece of an apple mouth-to-mouth through the bars of the cage to his female neighbor (Schaller 1963).

The male–female food transfer evolves in the species along the possibility of choosing a partner by the female. Sharing food within the gender is associated with forming a coalition. These phenomena are statistically related to each other (Jaeggi and Van Schaik 2011). Fruit located in high tree branches is important in the chimpanzees’ diet. This type of fruit, difficult to obtain and therefore attractive, in the hands of a male can arouse the females’ interest in it. Gorillas lead a terrestrial life and feed on leaves and rhizomes, so that both genders can easily harvest from the ground. Gorilla males neither establish a coalition aimed at group competition for the females, nor they allow female choices, so food sharing does not occur in this species. However, when a gorilla male does not claim rights to a female, it gives her food.

Among primitive tribes, a phenomenon of sharing meat between related and unrelated members of the community is popular (Kaplan et al. 1985; Le Jeune 1897). This may result directly from the inability to store it and the unpredictability and relative rarity of success on the hunt of individual hunters. Researchers state that in societies that collectively divide meat, better hunters have more children. They indicate that there are two reasons for this relationship. First, more effective hunters are more desired by women, which makes them have relatively easier access to extramarital sex. Secondly, tribe members are more willing to look after the offspring of better hunters. Hunting skills, in connection with the habit of meat sharing, do not contribute to the multiplication of men’s goods, but decide about their social position, which affects both access to women and the quality of care for their offspring (Kaplan et al. 1984; Kaplan and Hill 1985; Hill and Kaplan 1988). In other words, it can be claimed that “the hunter hunts for lovers, not for wives.”

Research on the advantages appreciated by American women from permanent and fleeting partners has shown that four advantages are more highly valued among candidates for a fleeting contact. These are: spending a large amount of money on a woman from the beginning, giving her valuable gifts from the beginning, leading a sumptuous lifestyle and making generously his resources available to her. These four virtues are expected moderately from husbands and highly from partners of a fleeting relation (Buss and Schmitt 1993).

In matricentric human societies, like island Trobrianders, men bring their lovers various goods: food, tobacco and ornaments, and women agree on sexual relations as long as a stream of gifts flows. A woman may say, “*If you do not have a gift for me, then I do not agree.*” The reputation of a Trobriand man may suffer among the women if he refuses to give a gift to his lover, and as a result, his ability to obtain another one will decrease too (Malinowski 1929).

In Nayar’s polyandry relationships from southwest India, a girl married one man, but later, in fact, she had sexual relations with other men who were not related to one another. She lived alone and had relations with them in succession, with their consent. In connection with the matrilineal system of these people, children inherited from their mother’s brother. The whole burden of child care rested on its matrilineal relatives. However, when the woman became pregnant, she indicated a particular man as the father of the child and he was obliged to support her in upbringing (Malinowski 1929). Under such conditions, biological paternity was not very important and social paternity belonged to the man who paid the midwife for delivering a child. The “husbands” did not live with their “wives,” but with their maternal relatives whom they visited for the night. Each of the parties could end such a relationship at any time, without any special formalities. The visiting partner brought a small cash gift to the woman, while a more regular “husband” gave her (at the beginning of the relationship) some material for a skirt and then gifts on the occasion of three annual festivals (e.g., loincloths, Beretta leaves, Areca nuts, hair and bath oils and some types of vegetables). Not giving such a gift was a sign that the man wanted to end the relationship (Gough 1959).

Matricentric Mosuo people live near Lugu Hu Lake and in Sichuan in Southwestern China. Every household consists of 8 to 20–30 members and these are multigenerational families, making their living by cultivating land. The relationship between a woman and a man is that they permanently live in their family homes, and the man accepted by the woman stays with her for the night and then returns home. The institution of the father is not known in the Mosuo people. Children born of such a relationship belong to the woman and they inherit from her, take her surname and are brought up by her family. The role of the father is performed by their uncles. All children in a given family are brought up together. Parental separation does not affect their situation. Parents do not have any economic ties. Mosuo’s “walking marriage” is based solely on love and mutual attraction. Acceptance of the man, as a son-in-law, usually takes place when the girl’s mother accepts customary gifts: tea, wine, clothes for the girl. The language of the Mosuo does not know such words as: war, jealousy, rape and murder (Mattison 2011).

A phenomenon of male–female food transfer present in the patriarchal chimpanzee allows dominated males, by establishing long-term relationships with the female, for a reproductive success: the female will agree on the estrus copulation. In exchange, bearing the full parental investment, she gets increased access to food. The bonobo females, constantly attractive and receptive, allow all the males to approach them through the entire menstrual cycle, which allowed matriarchy and thus the priority of the females in access to food. The male–female food transfer phenomenon is also significant for *Homo sapiens*. In societies where there are no conventional, long-term patriarchal marriages, where a woman chooses a sexual partner and is independent from him in an existential way, the transfer of resources to the sexual partner (the woman) is expected. In primitive communities, the women find it easier to make sexual contact with effective hunters from whom they get meat. Meanwhile, contemporary women from a monogamous American culture expect from their fleeting partners intense transfer of money. In matricentric human societies, women are not dependent on men’s resources, but they also expect support from them.

## Male and female sexual jealousy

The analysis of jealousy carried out by Symons (1979) and Daly et al. (1982) provides the main theoretical basis for the evolutionary analysis of the jealousy. Sexual jealousy is conceptualized as a functional emotion, whose basic purpose is to preserve the bonds of the precious relationship (Buss 2000, 2013). Dozens of empirical studies on the psychology of the jealousy indicate that men and women do not differ in the frequency or level of the jealousy they experience. Male jealousy, compared to the female one, seems to be more sensitive to signals of sexual infidelity. Women’s jealousy, on the other hand, seems to be relatively more sensitive to signals of emotional unfaithfulness (Buss 2013).

The hierarchy of the females that conditions access to food is present in the chimpanzee species. The females are aggressive toward new immigrant women and even kill newborn babies of community members (Pusey and Schroepfer-Walker 2013). Woman aggression is directed mainly against other women and generally causes minor injuries. The most common victims are relatives and rivals in sex and marriage. The aggression is often a means of competing for people or food products (Burbank 1987). The bonobo females differ from the chimpanzees and from women; they work together to dominate adult males in competition for food (Furuichi 1997; Vervaecke et al. 2000).

Table 2 presents factors generating the jealousy of a hominid female. Resources necessary for survival are divided into two groups: “unstable”—on a current basis derived from the natural environment (the open set), and “accumulated”—obtained by agriculture (the closed sets). The last line presents the expected level of the female jealousy of a male, determined on the basis of the level of factors generating this jealousy. The term “female jealousy of the male” and “male jealousy of the female” is understood here as imposing sexual restrictions on the opposite gender. Table 2 indicates that the level of the female jealousy of the male is closely related to the socio-environmental conditions in which she functions.

**Table 2:** Distribution of the hominid female jealousy of the male (Goodall 1986; Watts 1991; Stanford 1998; Kramer 2011; Malinowski 1929; Mattison 2011; Gough 1959; Hill and Kaplan 1988; Kano 1980; Jaeggi et al. 2013; Schaller 1963; Dixson 1981; Jaeggi and Van Schaik 2011; Kaplan et al. 1984; Buss and Schmitt 1993; Smuts and Smuts 1993)

Female♀	Apes			*Homo sapiens*		
Social conditions	Gorilla (*Gorilla*) One-male harem	Chimpanzee (*P. troglodytes*) Multi-male harem	Bonobo (*P. paniscus*) Matriarchy	Polygyny and monogamy (unstable resources in open set	Polygyny and monogamy (accumulated resources—in closed sets)	Matricentric polyandry
**Factors**						
Access to environmental resources:	Yes	Yes—hierarchic	Yes—females have priority	Partial—females do not get meat	Partial or no access	Yes
Access to preferred sexual partners:	Limited—except for access to the dominant male	Partial	Unlimited	Limited	Limited	Unlimited
Economic dependence on the male:	Absent	Absent	Absent	Absent –meat is consumed collectively	Present	Absent
Expected level of gifts from males:	None or low	Low	Low	Low	High	Low
Need for protection from the male against sexual aggression:	Permanent—during the estrus and against infanticide	Permanent—during the estrus and against infanticide	Absent	Permanent	Permanent	Female is protected by her brothers
Need for support from the male in the care for offspring:	Absent	Absent	Absent	Present	Present	Low level—the offspring of women are taken care of mainly by her relatives
Expected level of the female jealousy of the male:	Low	Low	Absent	Medium	High	Low

Factors significantly generating the female jealousy are underlined

Male sexual jealousy is a common function of the dominant male of chimpanzees, gorillas and other herding mammals. Chimpanzee dominant males show jealousy mainly of females in the estrus. Sometimes they direct aggression to lower-ranking males, but sometimes also to a female for her sexual relations with others (De Waal 1982). In patriarchal polygyno-monogamous human societies, a man can have access and a monopoly usually on a relatively small number of women in a given period of time (often only one), through which he can pursue his reproductive success. A similar situation occurs in the dominant males of the chimpanzees or gorillas who have access to and monopoly on a relatively small number of the females currently signaling ovulation. In contrast, bonobo males have free access to all the females, as there are no dominant males there and the females permanently signal the state of ovulation. The woman is also permanently attractive, so in the eyes of the men she is constantly in a potential ovulation state. Therefore, the jealousy of the man who is holding a monopoly on the woman will also be permanent.

The hypothesis of the certainty of fatherhood, as the source of the male jealousy presented by, for example, Alexander and Noonan (1979), is difficult to accept. In cases of sexual behaviors such as inter-marital infidelity, rape, war rape, group sex, in matricentric human societies, the bonobos and in the case of the dominated chimpanzee and gorilla males, the male jealousy is absent. It is also difficult to assume that in the course of evolution, fatherly investments would be the cause of the need to increase this certainty through the jealousy. In primitive communities, especially men with a high social position, devote little time to dealing with their own children (having the largest number of them) (Hewlett 1991).

Table 3 contains factors generating the male jealousy in modern hominids as a function of their socio-environmental conditions. The last line shows the expected level of the male jealousy, determined on the basis of the level of factors that generate this jealousy. Based on the cases of both chimpanzee species and the human matricentric societies, it is assumed that just limitation in access to the females causes a high level of the male jealousy of the female.

**Table 3:** Distribution of the hominid male sexual jealousy (Goodall 1986; Watts 1991; Stanford 1998; Kramer 2011; Malinowski 1929; Mattison 2011; Gough 1959; Hill and Kaplan 1988)

Male♂	Apes			Homo sapiens		
Social conditions	Gorilla (*Gorilla*) One-male harem	Chimpanzee (*Pan troglodytes*) Multi-male harem	Bonobo (*Pan paniscus*) Matriarchy	Polygyny and monogamy (unstable resources in open set)	Polygyny and monogamy (accumulated resources—in closed sets)	Matricentric polyandry
**Factors**						
Access to females	Limited	Limited	Unlimited	Limited	Limited	Facilitated
Level of investments directed to the female	Absent or low	Low	Low	Low	High	Low
Level of investments directed to offspring	Absent	Absent	Absent	Low	High	Low
Expected level of the male jealousy of the female	High (concerns the dominant male)	High (concerns the dominant male)	Absent or low	High (concerns the male claiming rights to the female)	Very high (concerns the male claiming rights to the female)	Low

Factors significantly generating the male jealousy are underlined

## Hominid sexual nature

Based on the features of the modern hominids discussed above, analogous social features of their common ancestor were extracted (Table 4). These features were then treated as the basis on which the construct of the “sexual nature of the hominids” was built.

**Table 4:** Social features of the hominids conditioning their sexuality (Jaeggi and Van Schaik 2011; Savage-Rumbaugh and Wilkerson 1978; Smuts and Smuts 1993; Watts 1989; Lancaster and Lancaster 1983; Jaeggi et al. 2013; Gomes and Boesch 2009; Basson 2001)

Feature	Female/male				
	*Homo sapiens*	Chimpanzee (*P. troglodytes*)	Bonobo (*P. paniscus*	Gorilla (*Gorilla*)	Last common ancestor
Social system	Mainly patriarchy, matricentrism	Patriarchy	Matriarchy	Patriarchy	Patriarchal social structure
Community	Heterogeneous	Heterogeneous	Heterogeneous	Homogenous	Heterogeneous community
Copulation periods (female sexual attractiveness)	Perovulatory; permanent attractiveness	Perovulatory	Permanent	Perovulatory	Perovulatory sexual behavior
Parental investments	Present/present and absent^a^	Present/absent	Present/absent	Present/absent	Females bear all parental investments
Male–female food sharing	Present	Present—for building long-term relationships	Present—male shares food with a seducing him female	Absent in the wild; present—if a female doesn’t belong to a male	Males share food with females

Information given by the authors is written in an extended text

^a^Data from the Central Statistical Office show that in Poland men make up approx. 96% of the total child support they are required to pay, of whom approx. 80% do not pay the granted support

The modern hominids function in various social structures: patriarchal and matriarchal. This is why it is difficult to define their “sexual nature.” The following methodology was used to determine the “sexuality cores” of the hominid male and female: it was based on the list of selected features of four hominid species, the presence of these features was determined in LCA. Then, by eliminating the influence of the patriarchal social structure on these features, their presence was defined as the “sexual cores” of the hominid male and female.

### Hominid female’s “core of sexuality”

Sexual behavior of the hominids, seen in the context of the degree of access restrictions for the females, allows to define the “cores of sexuality” specific to them. The cores are male and female sexual functions that are independent of the environmental influences and common for all social hominids. These functions incline the hominids to maximize their reproductive success. The “sexuality core” of the hominid female, as independent of the social structure, was extracted on the basis of the comparison of the selected features (Table 5).

**Table 5:** The “sexuality core” of the hominid female (Dixson 1998; Basson 2001; Wrangham 2001; Buss and Schmitt 1993; Stoinski et al. 2009a, b; Hauser 1990; Clay et al. 2011; Nadler 1976; Masters and Johnson 1966; Thornhill et al. 1995)

Feature	Female					
	*Homo sapiens*	Chimpanzee (*P. troglodytes*)	Bonobo (*P. paniscus*)	Gorilla (*Gorilla*)	Last common ancestor	Core of sexuality
Sexual preferences	Present—selectivity	Present—selectivity	Generally absent—possible promotion of selected males in the estrus season	Present—selectivity	Present—selectivity	Presence of sexual preferences—selectivity
Determinant of attractiveness of the opposite gender	Social status, lack of sexual aggression, gifts, physicality	Social status, food sharing	Lack of data	Social status	Social status, food sharing	Presence of males’ attractiveness features preferred by females
Need to protect against sexual aggression	Present	Present	Absent—females are sexually available for the entire period of the menstrual cycle	Present	Females need to be protected against sexual aggression	Need for protection against sexual aggression
Sexual inclinations	Polyandrism	Polyandrism	Polyandrism	Polyandrism	Polyandrism	Polyandrism
Sexual drive (proceptivity)	Perovulatory	Perovulatory	Permanent	Perovulatory	Perovulatory	Perovulatory sexual drive
Copulation calls	Present	Present	Present	Present	Present	Presence of copulation calls
Multiple orgasms	Present	Reaches orgasms	Reaches orgasms	Unknown	Reaches orgasms	Possibility of reaching multiple orgasms
Expected level of jealousy of the male	Depends on social structure^a^	Low	Absent	Low	Low	Low level of jealousy of the male

Information given by the authors is written in an extended text

^a^High level of the woman jealousy of the man—if the woman is dependent on care of one partner and needs his protection (in the patriarchal social structure); low level of the woman jealousy of the man—if the woman is protected and economically independent, if she has an access to multiple partners and their resources (e.g., the matricentric Mosuo people)

**Fig. 1:**
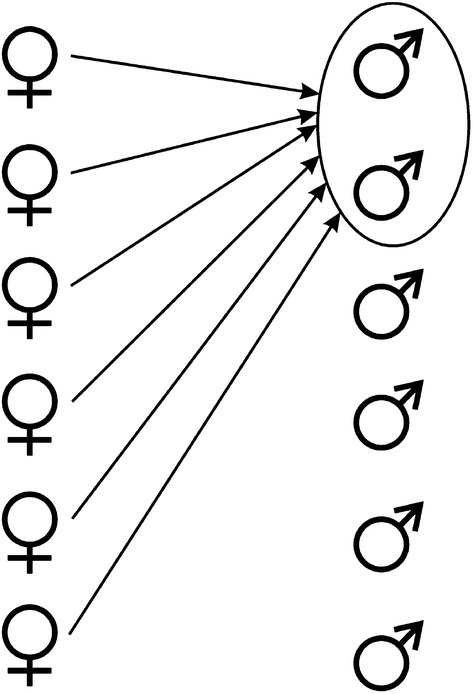
Selective polyandry

On the basis of Tables 4 and 5, it can be assumed that the hominid female tends to achieve full sexual freedom, “selective polyandry” (Fig. 1), being in safe living conditions. An essential condition ensuring the sexual freedom for her is a free choice of sexual partners and time of sexual intercourse. The quality of the father is crucial for her because of the parental investment that she incurs due to pregnancy and lactation and due to the reproductive success of her genetic line in the future.

### Hominid male’s “core of sexuality”

Features of the species on the basis of which the “sexuality core” of the hominid male was determined are presented in Table 6.

**Table 6:** The “sexuality core” of a hominid male (Dixson 1998; Wrangham 2001; Mosher and Abramson 1977)

Feature	Male					
	*Homo sapiens*	Chimpanzee (*P. troglodytes*)	Bonobo (*P. paniscus*)	Gorilla (*Gorilla*)	Last common ancestor	Core of sexuality
Sexual preferences	Absent—sufficiently intensive simulation of estrus activates the male sexual behavior	Absent	Absent	Absent	Absent	Lack of sexual preferences—signs of estrus activates male sexual behavior
Determinant of attractiveness of the opposite gender	Permanent attractiveness–simulation of estrus	Estrous swelling	Permanent estrous swelling	Proceptivity;	Estrous swelling	Signs of fertility are determinant of attractiveness
Sexual inclinations	Promiscuity	Promiscuity	Promiscuity	Promiscuity	Promiscuity	Promiscuity
Sexual drive	Permanent	Permanent	Permanent	Permanent	Permanent	Permanent sexual drive
Presence of sexual jealousy	Present/absent^a^	The dominant male is jealous of his harem; other males—no	Absent—females are available	The dominant male is jealous of his harem; other males—no	The dominant male was jealous of his harem; other males—no	Lack of the sexual jealousy

Information given by the authors is written in an extended text

^a^Present—under conditions of limited access to the females (in patriarchal social structures); absent—under conditions of availability of the females (e.g., war rape)

**Fig. 2:**
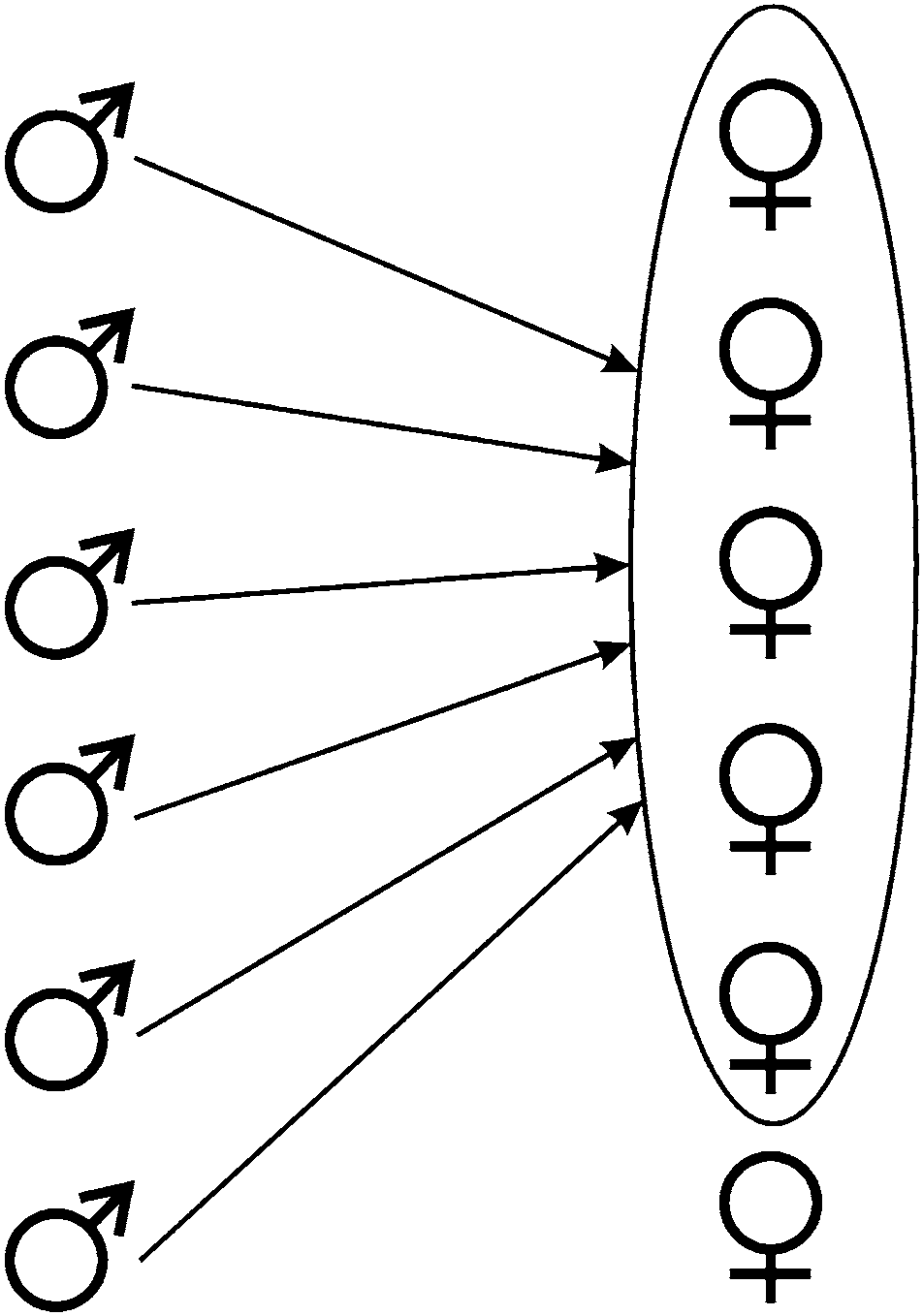
Tolerant promiscuity

Table 6 reveals that the “tolerant promiscuity” (Fig. 2) will be sexually comfortable for the male: free access to sexually attractive females and relationships with them in the conditions of sperm competition. The bonobo females adapted to that need. They gave the males sexual fulfillment; however, the cost to the males was resignation from dominating over the females. The hominid male does not need any choice of the female: the only criterion that the female should meet him again is her ability to stimulate him sexually—she must have clear enough signs of fertility.

## Discussion

### The gorilla phenomenon

Group violence against other communities (war) besides *Homo sapiens* is also present among patriarchal cooperating chimpanzees (*Pan troglodytes*) (Goodall 1986); a war provides dominated males with genetic advantages. Gorillas (*Gorilla*), however, do not cooperate, but they can also successfully support the patriarchal social structure (De Waal 1982).

The gorilla is the first clad which separated from the taxon leading to *Homo sapiens* and developed apomorphic features that are worth noting. Arboreality allows to acquire valuable fruit, while low parts of the bush mainly offer leaves and rhizomes (low-value food), for which the gorillas had to develop coprophagia (Akers and Schildkraut 1985), occupying these niches. Gorillas’ dominant males, increasing the body size, got rid of other males from the herd, because they were able to defend it alone against threats from the environment. Therefore, both adolescent daughters and sons were removed from the herd. After getting rid of internal rivals in accessing the females, they established a harem structure, reducing the need for sperm and thus the size of testicles: the gorilla male has the smallest testicles among the hominids (Harcourt et al. 1981). Taking into account low-value nutritional conditions, it is possible that for these primates, investing in the growth of the male’s mass and functioning in small, harem groups, turned out to be more energy-effective than in-group competition for the females with other males in the heterogeneous herd.

Sexual aggression and infanticide among mountain gorillas (*Gorilla beringei beringei*) are widespread (Harcourt and Stewart 1987), which links them with the chimpanzees. Infants living in the male leader group are rarely killed, while almost half of all the infants who leave it are killed shortly thereafter by other males. These data support the hypothesis that the murder of the offspring directs the selection responsible for the group life of the gorillas (Watts 1989; Wrangham 1979, 1982, 1987).

The sexual aggression and infanticide could contribute to the disappearance of estrous swelling in gorilla females. Visual estrus signaling generates inter-male competition, hence uncertainty of fatherhood. In the case of a harem herd consisting of one male reproducer and his females, female infidelity had to end with the murder of her young, committed by a dominant male. Therefore, it was in the interest of the females to maintain fidelity to the dominant male and her proceptive behavior turned out to be enough to arouse him sexually.

Thus, it can be assumed that the hominid LCA (like chimpanzees) had functioned in the terrestrial and arboreal environment in the patriarchal social structures, and their females were receptive in the perovulatory periods and signaled the estrus with anal–genital swelling. The presence of swelling indicates heterogeneity of the community—the presence of other males in the herd next to the dominant one (Table 4).

### Hominid jealousy

In human matricentric societies, where a woman resides in her mother’s home and this house protects her and her offspring, where there are no conventional marriages, and in the case of matriarchal bonobos—the female jealousy is not clear. Women in the patriarchal structures are jealous of the men to whom they allow sexual access in exchange for the access to the resources that these men have, while the females of the patriarchal chimpanzees compete with other females for access to environmental resources (Table 2). Therefore, it can be concluded that the jealousy of the hominid female is directly linked to the patriarchal social structure, and thus derives from interpersonal competition for the resources. The chimpanzee female competes for the resources but not for the males, because they do not have them. In the case when the female is forced to derive the resources or other benefits from the male, her jealousy takes the form of the jealousy of the male and manifests itself in the aggression directed against him and the females threatening her relationship. That is why the institution of the first wife (having most rights) is popular among the polygynous *Homo sapiens* societies.

Benefits that a woman can derive from a man can, of course, be the resources that he has but also protection against sexual aggression and his help in raising her children and in the household. When the woman establishes sexual relations with the man who fulfills her preferences, then the benefit that she obtains from him will also be her sexual gratification. In patriarchal societies, the woman has extremely limited access to sexual partners. Directing the attention of her man to other women threatens not only her insufficient sexual satisfaction, but even its loss; her jealousy takes the form of strictly “sexual jealousy.”

Under patrilocal conditions, the female without any relatives in her community can only count on the male to whom she belongs. Stopping the male from directing his energy in search of new sexual objects allows him to redirect this energy to protect and care for a jealous female and her offspring. The jealousy of the male who does not meet female’s sexual preferences, but provides protection against sexual aggression and provides support and protection (e.g., during wars) might ensure the survival of the female and her offspring. In the Yanomamö tribe, if a woman does not have male protection, she is at high risk of rape and beating; her children may also be killed (Biocca 1970). Therefore, the presence of the female jealousy of the male suggests that this line has evolved mainly in the patriarchal and thus patrilocal environment, where the females without the support of relatives fought for survival and reproduction in the company of the male to whom she belonged.

The presence of the male jealousy only in sexual relations, in which the man claims the rights to the woman, the low level of man’s investment and involvement in raising children, and his preference, if there is such a possibility, of virgins for wives indicates that this jealousy is not related to the need for certainty of paternity that justifies investments in the offspring. However, these factors clearly indicate the man’s tendency to monopolize the sexuality of the woman. Under certain conditions, the man seems to seek to suppress the woman’s sexuality. These conditions are socially limited access to the females, present in all patriarchal primate structures.

Symons (1979) argues that the male sexual jealousy is more unchangeable and “obligatory” than the female one but suggests that the male sexual jealousy can be suppressed or inactivated in the context of polyamory. He suggests that this is possible because of male desire for sexual diversity. Having sexual access to other women in exchange, a given man will allow other men to have sex with his wife. It is worth adding here that in the conditions of polyamory, no man will feel the need to marry.

In a traditional patriarchal resource-accumulating community, the man is obliged to invest in his woman and her children because they are economically dependent on him. These investments will be an additional factor (not a source) heightening his sexual jealousy toward his woman—the man does not want someone else to meet his sexual needs through the woman he maintains; he does not want to invest in somebody else’s offspring and, at his own expense, increase the reproductive success of other men. In matricentric communities, where there are no conventional long-term marriages and therefore, access to the women is facilitated, and where the man does not invest heavily in either the woman or in his offspring, the male jealousy does not apply.

The above analysis indicates that the patriarchal social structures condition the escalation of the jealousy, in both the hominid female and male (Table 3). The dominance of the males entails the ownership of the females, so mutual free sexual access is limited. Limited access to sexual partners is a sufficient factor to induce a high level of the jealousy in the males claiming rights to the females. The female jealousy of the male, in the case when she has free access to environmental resources, would not be justified.  However, the male becomes substantially conditions the female’s being due to such factors as: economic dependence on the male, a need for his protection and support, benefits received from the males in the form of valuable gifts. Under such conditions, it is in the best interests of the female to cut off the male from all other females—the tool that the female will use is sexual jealousy. Facilitated access to the females (as in matricentric human communities) significantly reduces, and promiscuity of the females (bonobo) even eliminates the sexual jealousy, both male and female.

### Hominid “cores of sexuality”

Assuming no social structure, the hominid females’ “selective polyandry” would be estrous sexual relationships with more than one male of her choice (preferably at the same time), where each of them will be attractive enough to lead her to an orgasm (Table 5). This will allow sperm rivalry of several high-quality males in her reproductive tracts, giving her a chance for the highest quality offspring. Out-estrus sexuality of the hominid female allows her to select the males during non-ovulatory periods. She continues to “flirt” with the male, who activates her desire. Through the “flirt” the female chooses sexual partners, and by loud vocalizations during copulation, she stimulates them sexually, informs them about the state of her ovulation and about their ability to take part in exciting copulation under the sperm competition conditions. The female may show a low level of jealousy of the males who, when flirting, give her presents.

The chimpanzee males, who are not dominants, give females food and build relationships with them in this way. Male–female food sharing among gorillas in the wild has not been found. A gorilla dominant male has no competition in the herd and has a monopoly on the females, and he does not have to seek their favors. However, in captivity, when the female was separated from the male by bars (so she did not belong to him), he gave her an apple. American women (probably not only them), expect valuable gifts from candidates for a fleeting contact. In the case of the women from the matricentric societies, the end of the inflow of gifts from the man means the end of mutual relations. Therefore, in conditions of independence from the male, the hominid female expects male’s gifts to improve the quality of her life and facilitate her reproductive success.

The female Coolidge effect was found in rodent species in which the females benefited from copulation with many males (Lisk and Baron 1982; Lester and Gorzalka 1988; Ventura-Aquino and Fernández-Guasti 2013). Therefore, the hominid female should also be concerned with the Coolidge effect. The more high-quality males she flirts with, the more gifts she gets from them and the more her quality of life increases. Cost-effectiveness of polyandrous behavior of the hominid female is matched with her multiple orgasms and copulatory vocalizations. If the hominid female did not concern the Coolidge effect, the presence of women’s out-estrus “triggered” sexuality would not be justified. The woman would also be deprived of the possibility of semen selection. However, she is equipped with appropriate tools such as orgasms. Being limited to one male significantly reduces the number of gifts received by the female, and when he claims to own her, she does not receive gifts at all—as in the case of the gorillas.

A hominid male “core” has been identified as the “tolerant promiscuity” (Table 6). Tolerability reduces male’s reproduction chances by the sperm competition but it gives him exciting promiscuous copulations (for men it is more attractive to participate in a promiscuous copulation than in a sexual act of a heterosexual couple) (Mosher and Abramson 1977). Group sex participants (swingers) use rules to create more freedom and safety to act (Harviainen and Frank 2018). Therefore, the hominid male will be inclined to both: monogamous sex—increasing his reproductive chances, and multi-male sex—eliminating the need for inter-male aggression and giving him more satisfaction. However, it should be noted here that the hominid male is inherently jealous of the females to which he claims rights: the *LCA* has been functioning in the patriarchate and thus in conditions of limited access to the females.

The hominid male will be also inclined to both, multi-male and multi-female group sex. The multi-male group sex will stimulate his testicles to produce large amounts of sperm for rivalry in the female reproductive tract, as it is the case of the chimpanzee or bonobo (Møller 1988). Copulation with multiple females at the same time, analogically to a multi-male act, will be a strong sexual stimulus, causing testicles to produce semen, which he has to provide all the females with. The more females in the act, the higher the degree of excitement of the male.

### “Cores of sexuality” and the patriarchate

Analyzing contemporary views on sexual strategies for human mate choice Easton et al. (2015) divide both women’s and men’s strategies into short-term and long-term mating. The “sexuality cores” designated here do not indicate any hominid long-term mating tendency. This strategy is associated with limiting sexual freedom of the partner, so it is not a comfortable strategy and cannot be a part of “human nature.” This strategy neither occurs in bonobos nor in matricentric *Homo sapiens*. Among chimpanzees, there is fierce competition for access to fertile females, while among gorillas neither gender chooses a partner. Long-term mating is therefore a strategy only characteristic of patriarchal *Homo sapiens* with long-term property rights on the sexual partner and extremely limited mutual choice of partners that results from these rights. Therefore, this strategy is only the outcome of restrictive *Homo sapiens* patriarchal social conditions and is applicable at the expense of giving up meeting psychological and biological needs of both genders.

If hypothetical hominid species gained free access to the environmental and sexual resources it needed, then both genders would use only their “cores of sexuality”: the males would be tolerant and promiscuous, and the females would choose the best of them. However, these “cores” do not coincide with each other and create a social conflict in the form of the males rejected by the females.

A hypothetical, primary heterogeneous community of the hominids, in which no social structure yet exists, will be considered below. The males are not yet able to cooperate, the females clearly signal ovulation, both genders have free access to food, and both have the aforementioned “sexuality cores” (in the form of “tolerant promiscuity” of the male and “selective polyandry” of the female). By adopting an early stage of social evolution, it is not a very numerous herd. Both young males and females migrate between flocks. During the estrus periods, the females will seek to mate only with the most attractive males. So a small group of the preferred and a larger group of the rejected males by the females will be formed. All the males are promiscuous and they have high sexual needs, so they will demand copulation. The refused males will begin to apply sexual coercion to the females and aggression toward the privileged males. Competition for access to the females under the rape conditions will begin. In this situation, the females will seek protection of the preferred males. These males are attractive to the females—so they are also healthy and strong. Therefore, they will restrict access to the females from the unwanted males. Under conditions of competition for the females, a discussion about mutual inter-male sexual tolerance is no longer valid. In a herd with not many members, the strongest male (unable to cooperate) will protect all the females in the estrus, and thus will gain a monopoly on access to them, as it is the case of gorillas. In numerous human and chimpanzee communities, patriarchy is maintained due to the cooperation of the males and thus to the male hierarchy.

The above brief analysis of the “primary hominid” behavior reveals that having particular “cores of sexuality,” under conditions of periodical estrus signaling, condemns the hominid females to sexual aggression and generates a need for their protection. Therefore, the whole hominid family is condemned to a patriarchal social structure and, as a result, to sexual coercion and adultery which allows the females to make a choice. Patriarchy, combined with the selectivity of the females, is the cause of extreme sexual restrictions for the males. This paves the way for searching other sexual tension unloading channels such as war rapes, prostitution, harem tendencies. Polygyno-monogamous tribal patriarchy, in conditions of the permanently simulated estrus, will also generate marital rape and marital prostitution.

The exceptions from the patriarchal fate are: the bonobo, whose females, resigning from selectivity, satisfy the promiscuous needs of the males, so they do not compete for access to them; the *Homo sapiens* matricentric communities, characterized by the lack of males’ monopoly on the females and, as a result, by facilitated access to them. The females from both species simulate the state of permanent ovulation, which suggests that estrus simulation is a necessary condition but not enough to reduce a patriarchal social system. *Homo sapiens* mostly functions in patriarchal structures; therefore, simulation of the permanent ovulation, without stretching the female’s sex drive for the entire menstrual cycle, does not guarantee social structures without the male hierarchy.

The female’s needs are mutually exclusive in patriarchal structures. The need for care creates the sexual monopoly on the female in care of the male. This monopoly is the cause of her limited availability and thus, of her limited sexual freedom. Patriarchal structures also restrict women’s free choice, limiting it to infidelity. Meeting sexual needs of both genders in an optimized way provides matricentric social structures of *Homo sapiens*, where her relatives take care of the woman and her children and thanks to that, she can enjoy sexual freedom. From the males, she only expects physical attractiveness, gifts and lack of aggression. Matricentry does not provide the men with conditions for total promiscuity because giving them facilitated access to the women imposes on them meeting women’s preferences. However, the lack of a male hierarchy causes that these communities break down under the influence of aggressive organized actions of patriarchal groups, focused on the proprietary acquisition of the women.

## Summary

The analysis of the hominids sexual behavior carried out in this paper indicates that their social structures manifest various uses of socio-environmental conditions to meet their biological, existential and emotional needs. This behavior is based on the common to all the hominids “cores of sexuality.” These “cores” are evolutionary constant psychosomatic mechanisms that direct the organism at the maximization of its reproductive success. Due to a huge disparity regarding parental investments between the hominid males and females, these “cores” differ from each other.

The results of this analysis correlate with the Trivers (1972) theorem. However, it has been also shown here that the “tolerantly promiscuous” hominid male, by his very nature, does not choose female partners at all. He is satisfied if the female is able to sexually stimulate him. The hominid male (including the man) does not show paternal instincts, so it does not make him any difference which female he fertilizes—each of this male’s offspring will inherit his tendency to opportunism. Under conditions of free access to the females, the hominid male will not compete for them, but he will be inclined to (exciting for him) copulations in the conditions of sperm rivalry and high demand for sperm. In turn, the possibility of choice is a key condition for the hominid female: the female chooses the best males from those available (“selective polyandry”) and their sperm will compete in her reproductive tracts for access to the ovum. She will bear a full parental investment in the future.

The natural (free) mating cycle of a *Homo sapiens* female consists, therefore, of the following stages:

1. Outside the ovulatory period, following the “triggered sexuality,” from her surroundings the female chooses the males who arouse her desire (also by means of gifts) and by flirting, she informs them about her potential sexual accessibility;
2. In the first phase of the ovulation period, the woman may have a tendency to perform a mating dance, announcing the surrounding men her estrous state. A spontaneous mating dance of the woman was observed on the 12th day of her menstrual cycle. It was performed publicly, in conditions of reduced sexual restrictions (the authors’ own observation).
3. In the estrus phase, the female copulating with the male of her choice loudly vocalizes, invoking the other males befriended with her and by this, stimulating her partner (group copulation with invoked males is more exciting to him than monogamous mating, loud vocalization also “raises” the social status of the copulating male). By vocalization, the female increases the vigilance of “her” males and therefore, also her own safety; unwanted males will not be allowed to her by the presence of the chosen ones;
4. During copulation, the female using the potential of multiple orgasms achieves the orgasm with each of subsequently selected males, sucking their sperm into the genital tract. In such conditions, the ovum is fertilized by the “best of the best” spermatozoid.

By the presence of out-estrus sexuality, the hominid female, while selecting the males, can be guided by the “triggered sexuality”—she continues flirting with the male who activates her desire, also by bestowing her. The women’s “triggered sexuality” should therefore be treated as an evolutionary tool inherited from common ancestor: the females from all four hominid species also copulate in out-estrus periods. This is used to determine the quality of the males, their selection, and it is aimed at selecting those, who will be able to give her one of the orgasms during multi-male estrous copulation.

Free multi-male group sex as a function of sexual selection seems to be the basis for the effective evolution of the hominids. The institution of marriage, both traditional (polygynous) and Euramerican (monogamous), blocking the natural polyandrous needs of the women, to a large extent blocks the influence of sexual selection in the species *Homo sapiens*. If the women had existential security (e.g., thanks to relatives) and sexual freedom (as in matricentric communities), they could realize their “core of sexuality,” and for each of their pregnancies they would acquire the best genetic material.

The hominid females determine the social structures through their sexual selectivity or opportunism, proceptivity, and the way of estrus signaling (continuous or periodic). The need for selection of the hominid female periodically signaling ovulation determines the patriarchal social structure (the common chimpanzee, the gorilla). The need for selection of the females feeling perovulatory desire and simulating the permanent state of ovulation, in conditions of wars and thus, of the male hierarchy, also results in the patriarchal structure (*Homo sapiens*). On the other hand, the absence of wars and thus, of the male hierarchy (the hierarchy falls as a result of adultery of the females) allows such females to create structures based on their kinship (*Homo sapiens*). Sexual opportunism of the females being permanently receptive, proceptive and simulating the permanent state of ovulation, by breaking the male dominance, creates a matriarchal type of the community (the bonobo) (Table 7).

**Table 7:** Hominid social structures and the females’ sexuality

Feature	Female sexual preferences	Ovulation signaling (sexual attractiveness of the females)	Female sexual drive	Group aggression of males (war)	Social structure
Species					
Gorilla (*Gorilla*)	Present	Proceptive behavior during estrus	Perovulatory	Absent—lack of male cooperation	Patriarchy
Bonobo (*P. paniscus*)	Absent (opportunism)	Simulators of the permanent estrus	Permanent	Absent—lack of male hierarchy	Matriarchy
Chimpanzee (*P. troglodytes*)	Present	Estrous anal–genital swelling	Perovulatory	Present	Patriarchy
*Homo sapiens*	Present	Simulators of the permanent estrus	Perovulatory	Present	Patriarchy
				Absent—lack of male hierarchy	Matricentrism

If the women were not only permanently sexually attractive, but also stretched the sexual drive for the entire menstrual cycle, as the bonobo females do, then undoubtedly, in the species *Homo sapiens*, neither marriage nor war would appear. Moreover, women’s estrous sexual desire indicates that in the history of the hominin line evolution, a matriarchal structure, equivalent to that of bonobo’s, could not have taken place.
